# Supplementary material for: Perceptions of social media challenges and benefits during the Covid-19 pandemic: Qualitative findings from a cross sectional international survey
Source: PLOS Glob Public Health. 2023 Jan 10;3(1):e0001463. doi: 10.1371/journal.pgph.0001463 (PMC10022235; doi:10.1371/journal.pgph.0001463)
Supplement: Attachment — Submitted filename: Response to reviewers PLOS Global Health (1).docx [file pgph.0001463.s002.docx]

Dear Editors and Reviewers

Thank you for your time and suggestions on how to improve the manuscript titled ‘Perceptions of social media challenges and benefits during the Covid-19 pandemic: qualitative findings from a cross sectional international survey’.

We have addressed all Editors and Reviewers comments and we think the changes significantly improved the manuscript. We have provided responses in the table below to each individual suggestion. Two corrected versions of the manuscript have been submitted. One with tracked changes to show where the changes were made and one clean version as requested.

We have also adhered to the Journal Requirements below:

1. Data available statement needs to be updated. *A minimal anonymized data set has been uploaded as Supporting Information files, as requested.*

2. Please send a completed 'Competing Interests' statement, including any COIs declared by your co-authors. If you have no competing interests to declare, please state "The authors have declared that no competing interests exist". Otherwise please declare all competing interests beginning with the statement "I have read the journal's policy and the authors of this manuscript have the following competing interests:" *Completed*

3. Please amend your detailed Financial Disclosure statement. This is published with the article. It must therefore be completed in full sentences and contain the exact wording you wish to be published. *Completed.*

i. Please clarify all sources of funding (financial or material support) for your study. List the grants (with grant number) or organizations (with url) that supported your study, including funding received from your institution. 
ii. State the initials, alongside each funding source, of each author to receive each grant. *Completed*.

If you did not receive any funding for this study, please simply state: “The authors received no specific funding for this work.” *Completed.*

| Editors and Reviewers comments | Authors responses |
| --- | --- |
| The paper reads well. However, it is unclear why you refer your study design as a mixed - method. It seems to me this was a survey whereby you had a mix of closed and open-ended questions. You conducted content analysis of the open-ended questions. That should be clarified. | Thank you for your suggestion. We have now corrected this to reflect your suggestion in the abstract and the method/design section in the main body (page 3 and 6). |
| **Reviewer #1:** This is a qualitative study to understand the effect and role of social media during the COVID-19 pandemic. The topic is important due to increased internet and social media use during the pandemic. Here is my comments  1/ Qualitative studies do not in general aim to generalize the results. The authors may want to justify the data collection methods. In fact, in depth interview to some of this participants may explain much further more. E.g. why some people felt more connected with social media while some others had increased anxiety. I suppose because it is online questionnaire, the answers will often be short and it is often not possible fully understand an individual story. I think individual interviews and focus groups may achieve this aim much better | Thank you for your comment. We have added this as a limitation to our design in the discussion section (page 23). |
| 2/ As the authors also mentioned that this is part of a mixed method study, how are the results "mixed"? at what level? how do the quantitatively and qualitative results interact? It is ok not to include all the results in one study (often due to limited word count) but the authors can show where we can find the mixed results | Thank you for your comment, and we refer to our previous response concerned with describing a ‘mixed methods’ study. We have added the information for the readers where they can access the findings for the quantitative part of the survey (page 6, reference 10) |
| 3/ Along the same thought, is triangulation possible? This may enhance the credibility of your results. | Thank you for your comment. In hindsight we acknowledge that the triangulation with quantitative data would have been useful. However, we also acknowledge that a lot of the quantitative data has been published in this area, and that there has been less qualitative data reported in the literature. Hence, we don’t intend to repeat this through triangulation. Instead, we consider that our paper with a qualitative focus adds something new to the literature. |
| **Reviewer #2**: I am glad to avail the opportunity to review this creative study entitled, “Perceptions of social media challenges and benefits during the Covid-19 pandemic: qualitative findings from an international survey.”  This study describes that since the beginning of the Covid-19 pandemic in January 2020 and the various national restrictions on social contact and travel in Europe, America and Australia, people became dependent on social media as a means for gathering information and a tool for staying connected to family, friends, and work. However, few cross-national qualitative studies have been done on the challenges and benefits of use of social media during the first wave of Covid-19. This cross-national study aimed to qualitatively examine the challenges and benefits of using social media during the Covid-19 pandemic outbreak.  I want to accept this study for publication. However, the authors need to revise it according to my suggestions. The title needs clarity with the design of the main study. I suggest authors revise their titles with better and suitable words. See the below-recommended studies to improve your TTLE and Abstract quality. | Thank you for your comment. We have added *cross-sectional* to the title. We kept the rest of the title as we think the title clearly describes the study (page 1). |
| Abstract First, I have some suggestions for the authors to enhance the quality of this innovative study. Please write a high-quality abstract, as it is the main door of the study. I suggest authors add Graphical Abstract in a meaningful way to reflect the whole idea. The abstract should be in a structured format. | Thank you for your comment. We have addressed your comment by changing the abstract (page 2 and 3). |
| Introduction section This section needs improvement. Please read these studies, revise your abstract, and cite them ithe introduction and literature part. Cite the suggested studies to improve the quality.  Su, Z., McDonnell, D., Wen, J., Kozak, M., Segalo, S., Li, X., Ahmad, J., Cheshmehzangi, A., Cai, Y., Yang, L., & Xiang, Y. T. (2021, Jan 5). Mental health consequences of COVID-19 media coverage: the need for effective crisis communication practices. Global Health, 17(1), 4. [https://checkpoint.url-protection.com/v1/url?o=https%3A//doi.org/10.1186/s12992-020-00654-4&g=ZGUzZmZiMGI1ZjIxY2IyYQ==&h=ZWMxZDI4OTg3MGEyYmFjZWU3NzY1ODcwNGMyZGNjZGYwNGFmMTEzMjMzNjZkMGU0ZWEyMTgzYTMyYWRlZjZlOQ==&p=Y3AxZTp1Y2xhbmxpdmU6YzpvOjI1M2Y4MjM2ODNkMDUyZWNiYTNlNjg1NDhjMjJlNzQ2OnYxOnQ6VA==](https://eur02.safelinks.protection.outlook.com/?url=https%3A%2F%2Fcheckpoint.url-protection.com%2Fv1%2Furl%3Fo%3Dhttps%253A%2F%2Fdoi.org%2F10.1186%2Fs12992-020-00654-4%26g%3DZGUzZmZiMGI1ZjIxY2IyYQ%3D%3D%26h%3DZWMxZDI4OTg3MGEyYmFjZWU3NzY1ODcwNGMyZGNjZGYwNGFmMTEzMjMzNjZkMGU0ZWEyMTgzYTMyYWRlZjZlOQ%3D%3D%26p%3DY3AxZTp1Y2xhbmxpdmU6YzpvOjI1M2Y4MjM2ODNkMDUyZWNiYTNlNjg1NDhjMjJlNzQ2OnYxOnQ6VA%3D%3D&data=05%7C01%7Cmariyana.schoultz%40northumbria.ac.uk%7C7e0cae9f2e964466f46908da97d9ad91%7Ce757cfdd1f354457af8f7c9c6b1437e3%7C0%7C0%7C637989258446931534%7CUnknown%7CTWFpbGZsb3d8eyJWIjoiMC4wLjAwMDAiLCJQIjoiV2luMzIiLCJBTiI6Ik1haWwiLCJXVCI6Mn0%3D%7C3000%7C%7C%7C&sdata=oZzVZzuNma9mjShiQNfOUytpZL%2Fu5%2FvLiZtVqXh8d3s%3D&reserved=0)  Azadi, N. A., Ziapour, A., Lebni, J. Y., Irandoost, S. F., & Chaboksavar, F. (2021). The effect of education based on health belief model on promoting preventive behaviors of hypertensive disease in staff of the Iran University of Medical Sciences. Archives of Public Health, 79(1), 69. doi:10.1186/s13690-021-00594-4  Maqsood, A., Rehman, G., & Mubeen, R. (2021, 2021/11/01/). The paradigm shift for educational system continuance in the advent of COVID-19 pandemic: Mental health challenges and reflections. Current Research in Behavioral Sciences, 2, 100011. [https://checkpoint.url-protection.com/v1/url?o=https%3A//doi.org/https%3A//doi.org/10.1016/j.crbeha.2020.100011&g=MDJlZTBjYmZiNjlmZGUxMg==&h=YjllNTcxMmFiODE5YTBmNGU3ZjIyMjZiMTZhNmYwZTMzZWE3NGU0OTBlMzAzNGI0ZmIyNGU5YThmYTRhOGJlYQ==&p=Y3AxZTp1Y2xhbmxpdmU6YzpvOjI1M2Y4MjM2ODNkMDUyZWNiYTNlNjg1NDhjMjJlNzQ2OnYxOnQ6VA==](https://eur02.safelinks.protection.outlook.com/?url=https%3A%2F%2Fcheckpoint.url-protection.com%2Fv1%2Furl%3Fo%3Dhttps%253A%2F%2Fdoi.org%2Fhttps%253A%2F%2Fdoi.org%2F10.1016%2Fj.crbeha.2020.100011%26g%3DMDJlZTBjYmZiNjlmZGUxMg%3D%3D%26h%3DYjllNTcxMmFiODE5YTBmNGU3ZjIyMjZiMTZhNmYwZTMzZWE3NGU0OTBlMzAzNGI0ZmIyNGU5YThmYTRhOGJlYQ%3D%3D%26p%3DY3AxZTp1Y2xhbmxpdmU6YzpvOjI1M2Y4MjM2ODNkMDUyZWNiYTNlNjg1NDhjMjJlNzQ2OnYxOnQ6VA%3D%3D&data=05%7C01%7Cmariyana.schoultz%40northumbria.ac.uk%7C7e0cae9f2e964466f46908da97d9ad91%7Ce757cfdd1f354457af8f7c9c6b1437e3%7C0%7C0%7C637989258446931534%7CUnknown%7CTWFpbGZsb3d8eyJWIjoiMC4wLjAwMDAiLCJQIjoiV2luMzIiLCJBTiI6Ik1haWwiLCJXVCI6Mn0%3D%7C3000%7C%7C%7C&sdata=YEvPhBQVTdJ5T0nUfeLcvRyAa5f4Oo2aPZqakEmUeZg%3D&reserved=0)  Abbas, J. (2020). The Impact of Coronavirus (SARS-CoV2) Epidemic on Individuals Mental Health: The Protective Measures of Pakistan in Managing and Sustaining Transmissible Disease. Psychiatr Danub, 32(3-4), 472-477. [https://checkpoint.url-protection.com/v1/url?o=https%3A//doi.org/10.24869/psyd.2020.472&g=MjU5ZjkxZjJlZTJhM2RhYg==&h=NTcyNjAzNWRkOTI1NGUwNWQ1YTc0MDQ5MGZiNmJkMmMyZDE3ZTdhMzhlMGMyYmVmYzk5YWFmOTA3N2VmN2FlMg==&p=Y3AxZTp1Y2xhbmxpdmU6YzpvOjI1M2Y4MjM2ODNkMDUyZWNiYTNlNjg1NDhjMjJlNzQ2OnYxOnQ6VA==](https://eur02.safelinks.protection.outlook.com/?url=https%3A%2F%2Fcheckpoint.url-protection.com%2Fv1%2Furl%3Fo%3Dhttps%253A%2F%2Fdoi.org%2F10.24869%2Fpsyd.2020.472%26g%3DMjU5ZjkxZjJlZTJhM2RhYg%3D%3D%26h%3DNTcyNjAzNWRkOTI1NGUwNWQ1YTc0MDQ5MGZiNmJkMmMyZDE3ZTdhMzhlMGMyYmVmYzk5YWFmOTA3N2VmN2FlMg%3D%3D%26p%3DY3AxZTp1Y2xhbmxpdmU6YzpvOjI1M2Y4MjM2ODNkMDUyZWNiYTNlNjg1NDhjMjJlNzQ2OnYxOnQ6VA%3D%3D&data=05%7C01%7Cmariyana.schoultz%40northumbria.ac.uk%7C7e0cae9f2e964466f46908da97d9ad91%7Ce757cfdd1f354457af8f7c9c6b1437e3%7C0%7C0%7C637989258446931534%7CUnknown%7CTWFpbGZsb3d8eyJWIjoiMC4wLjAwMDAiLCJQIjoiV2luMzIiLCJBTiI6Ik1haWwiLCJXVCI6Mn0%3D%7C3000%7C%7C%7C&sdata=0%2BLbcBg%2BnnpnGGBFI%2BokNAwbCOnp9vg6rfDW1APhwxc%3D&reserved=0) | Thank you for your comment. We reviewed our introduction and made changes according to your suggestions while incorporating some of the suggested literature that was compatible with our narrative (pages 5 and 6). |
|  |  |
| Literature In the emergence of the COVID-19 pandemic, healthcare systems have faced a tremendous pressure worldwide. This pandemic has affected all lifestyles. The study investigated an interesting research topic. The study offers interesting information and provides useful insight. I suggest authors cite latest literature related to COVID-19 pandemic effects to support the study. The suggested studies are;  Rahmat, T. E., Raza, S., Zahid, H., Mohd Sobri, F., & Sidiki, S. (2022). Nexus between integrating technology readiness 2.0 index and students’ e-library services adoption amid the COVID-19 challenges: Implications based on the theory of planned behavior. J Educ Health Promot, 11(1), 50. doi:10.4103/jehp.jehp_508_21  NeJhaddadgar, N., Ziapour, A., Zakkipour, G., Abolfathi, M., & Shabani, M. (2020, Nov 13). Effectiveness of telephone-based screening and triage during COVID-19 outbreak in the promoted primary healthcare system: a case study in Ardabil province, Iran. Z Gesundh Wiss, 1-6. [https://checkpoint.url-protection.com/v1/url?o=https%3A//doi.org/10.1007/s10389-020-01407-8&g=ZWIyOTFmNTMxY2ZiNzEzOQ==&h=NGVhZmZkNGMzMGJiYjJiYzk2YjcwM2YzMDRmZTU4MzdkNmE0OTc3ODZjZjRiYzFlOTI5MTVjNzI3NDlhMDQyNQ==&p=Y3AxZTp1Y2xhbmxpdmU6YzpvOjI1M2Y4MjM2ODNkMDUyZWNiYTNlNjg1NDhjMjJlNzQ2OnYxOnQ6VA==](https://eur02.safelinks.protection.outlook.com/?url=https%3A%2F%2Fcheckpoint.url-protection.com%2Fv1%2Furl%3Fo%3Dhttps%253A%2F%2Fdoi.org%2F10.1007%2Fs10389-020-01407-8%26g%3DZWIyOTFmNTMxY2ZiNzEzOQ%3D%3D%26h%3DNGVhZmZkNGMzMGJiYjJiYzk2YjcwM2YzMDRmZTU4MzdkNmE0OTc3ODZjZjRiYzFlOTI5MTVjNzI3NDlhMDQyNQ%3D%3D%26p%3DY3AxZTp1Y2xhbmxpdmU6YzpvOjI1M2Y4MjM2ODNkMDUyZWNiYTNlNjg1NDhjMjJlNzQ2OnYxOnQ6VA%3D%3D&data=05%7C01%7Cmariyana.schoultz%40northumbria.ac.uk%7C7e0cae9f2e964466f46908da97d9ad91%7Ce757cfdd1f354457af8f7c9c6b1437e3%7C0%7C0%7C637989258446931534%7CUnknown%7CTWFpbGZsb3d8eyJWIjoiMC4wLjAwMDAiLCJQIjoiV2luMzIiLCJBTiI6Ik1haWwiLCJXVCI6Mn0%3D%7C3000%7C%7C%7C&sdata=bTAYNPNao6CSujSuofTiYzDZAw5nu1mnmFWotXDRc0Y%3D&reserved=0)  Yoosefi Lebni, J., Moradi, F., Salahshoor, M. R., Chaboksavar, F., Irandoost, S. F., Nezhaddadgar, N., & Ziapour, A. (2020, Jul 2). How the COVID-19 pandemic effected economic, social, political, and cultural factors: A lesson from Iran. Int J Soc Psychiatry, 20764020939984. [https://checkpoint.url-protection.com/v1/url?o=https%3A//doi.org/10.1177/0020764020939984&g=ZTYyZDU2ODRmZmYyNmJhOA==&h=MTRlNzZkZTY3MDU0NmNlMjkxNzhkOWZhMGE0NzVhOGJhZDk3ZTg1OGIxMjYwYzMyMTkzZTkzMjRiNzY3ZjUwYg==&p=Y3AxZTp1Y2xhbmxpdmU6YzpvOjI1M2Y4MjM2ODNkMDUyZWNiYTNlNjg1NDhjMjJlNzQ2OnYxOnQ6VA==](https://eur02.safelinks.protection.outlook.com/?url=https%3A%2F%2Fcheckpoint.url-protection.com%2Fv1%2Furl%3Fo%3Dhttps%253A%2F%2Fdoi.org%2F10.1177%2F0020764020939984%26g%3DZTYyZDU2ODRmZmYyNmJhOA%3D%3D%26h%3DMTRlNzZkZTY3MDU0NmNlMjkxNzhkOWZhMGE0NzVhOGJhZDk3ZTg1OGIxMjYwYzMyMTkzZTkzMjRiNzY3ZjUwYg%3D%3D%26p%3DY3AxZTp1Y2xhbmxpdmU6YzpvOjI1M2Y4MjM2ODNkMDUyZWNiYTNlNjg1NDhjMjJlNzQ2OnYxOnQ6VA%3D%3D&data=05%7C01%7Cmariyana.schoultz%40northumbria.ac.uk%7C7e0cae9f2e964466f46908da97d9ad91%7Ce757cfdd1f354457af8f7c9c6b1437e3%7C0%7C0%7C637989258446931534%7CUnknown%7CTWFpbGZsb3d8eyJWIjoiMC4wLjAwMDAiLCJQIjoiV2luMzIiLCJBTiI6Ik1haWwiLCJXVCI6Mn0%3D%7C3000%7C%7C%7C&sdata=OOQh1TjjXynqWT2ldjYytmpVcCGmS6ILO%2BNG4LrRpgc%3D&reserved=0)  Su, Z., McDonnell, D., Cheshmehzangi, A., Li, X., & Cai, Y. (2021). The promise and perils of Unit 731 data to advance COVID-19 research. BMJ Global Health, 6(4). | Thank you for your comment. We reviewed our introduction and made changes according to your suggestions while incorporating some of the suggested literature that was compatible with our narrative (pages 5 and 6). |
| **Methods and Results** Please read the suggested studies and improve your method design and result sections. Cite these studies in methods and results to support the literature.  Li, Zhenhuan , Dake Wang, Kaifeng Duan, and R. Mubeen. 2021. "Social media efficacy in crisis management: Effectiveness of non-pharmaceutical interventions to manage the COVID-19 challenges." Front Psychiatry 12 (1099):626134. doi: 10.3389/fpsyt.2021.626134.  Aman, J., Nurunnabi, M., & Bano, S. (2019). The Impact of Social Media on Learning Behavior for Sustainable Education: Evidence of Students from Selected Universities in Pakistan. Sustainability, 11(6), 1683. [https://checkpoint.url-protection.com/v1/url?o=http%3A//www.mdpi.com/2071-1050/11/6/1683&g=MTU0NWQ3MjIwNjgwZDVmNw==&h=NGUxY2ZhM2E2NjIwZTRiMjk4ZWQzZjYwZGQ1NjdlOTQzYThmZWRhYmVlNDYxNGY3NDgyYWRjNTc1Y2YwYTlhYQ==&p=Y3AxZTp1Y2xhbmxpdmU6YzpvOjI1M2Y4MjM2ODNkMDUyZWNiYTNlNjg1NDhjMjJlNzQ2OnYxOnQ6VA==](https://eur02.safelinks.protection.outlook.com/?url=https%3A%2F%2Fcheckpoint.url-protection.com%2Fv1%2Furl%3Fo%3Dhttp%253A%2F%2Fwww.mdpi.com%2F2071-1050%2F11%2F6%2F1683%26g%3DMTU0NWQ3MjIwNjgwZDVmNw%3D%3D%26h%3DNGUxY2ZhM2E2NjIwZTRiMjk4ZWQzZjYwZGQ1NjdlOTQzYThmZWRhYmVlNDYxNGY3NDgyYWRjNTc1Y2YwYTlhYQ%3D%3D%26p%3DY3AxZTp1Y2xhbmxpdmU6YzpvOjI1M2Y4MjM2ODNkMDUyZWNiYTNlNjg1NDhjMjJlNzQ2OnYxOnQ6VA%3D%3D&data=05%7C01%7Cmariyana.schoultz%40northumbria.ac.uk%7C7e0cae9f2e964466f46908da97d9ad91%7Ce757cfdd1f354457af8f7c9c6b1437e3%7C0%7C0%7C637989258446931534%7CUnknown%7CTWFpbGZsb3d8eyJWIjoiMC4wLjAwMDAiLCJQIjoiV2luMzIiLCJBTiI6Ik1haWwiLCJXVCI6Mn0%3D%7C3000%7C%7C%7C&sdata=dxsDNJdBwD7%2BMy4DL74JC4QFXvHMQLvUQnHYKoiZVqE%3D&reserved=0)  Aqeel, M., Rehna, T., Shuja, K. H. (2022). Comparison of Students' Mental Wellbeing, Anxiety, Depression, and Quality of Life During COVID-19's Full and Partial (Smart) Lockdowns: A Follow-Up Study at a 5-Month Interval. Front Psychiatry, 13, 835585. doi:10.3389/fpsyt.2022.835585  Farzadfar, F., Naghavi, M., Sepanlou, S. G., Saeedi Moghaddam, S., Dangel, W. J., Davis Weaver, N., . . . Larijani, B. (2022). Health system performance in Iran: a systematic analysis for the Global Burden of Disease Study 2019. The Lancet. doi:10.1016/s0140-6736(21)02751-3 | Thank you for your comment. We have changed the methods section to bring more clarity as suggested. Please see methods section (page 6). The suggested literature here was not compatible with our design or methods so we couldn’t include it as reference. Moreover, as the results of the study are based on our own data collection, citations within the results section would be inappropriate. |
| Discussion is explained well. Check grammar errors in this section. I suggest authors cite latest literature related to COVID-19 pandemic effects to support the study. The suggested studies are;  Aqeel, M., Raza, S., & Aman, J. (2021). Portraying the multifaceted interplay between sexual harassment, job stress, social support and employees turnover intension amid COVID-19: A Multilevel Moderating Model. Foundation University Journal of Business & Economics, 6(2), 1-17. doi:https://checkpoint.url-protection.com/v1/url?o=https%3A//fui.edu.pk/fjs/index.php/fujbe/article/view/551&g=NWEzNTkyN2Q1MGVkZTZkNA==&h=MTQ1NzBmMjY2ZGE5ODZjZDE4ZWM4YjYzMTE3ZTJjMWZmYjYwZTc2Mjg2YjMyNmE3ZDJlNThjY2ExNTU2MzRmMQ==&p=Y3AxZTp1Y2xhbmxpdmU6YzpvOjI1M2Y4MjM2ODNkMDUyZWNiYTNlNjg1NDhjMjJlNzQ2OnYxOnQ6VA==  Abbas, J., Wang, D., Su, Z., & Ziapour, A. (2021). The Role of Social Media in the Advent of COVID-19 Pandemic: Crisis Management, Mental Health Challenges and Implications. Risk Manag Healthc Policy, 14, 1917-1932. doi:10.2147/RMHP.S284313  Geng, J., Ul Haq, S., Ye, H., Shahbaz, P., Abbas, A., & Cai, Y. (2022). Survival in Pandemic Times: Managing Energy Efficiency, Food Diversity, and Sustainable Practices of Nutrient Intake amid COVID-19 Crisis. Frontiers in Environmental Science, 13, 945774. doi:10.3389/fenvs.2022.945774  Yu, S., Draghici, A., Negulescu, O. H., & Ain, N. U. (2022). Social Media Application as a New Paradigm for Business Communication: The Role of COVID-19 Knowledge, Social Distancing, and Preventive Attitudes. Frontiers in Psychology, 13. doi:10.3389/fpsyg.2022.903082 | Thank you for your comment. We made changes according to the suggestion and grammar has been corrected. Please see changes (pages 19-22) |
| Conclusion  The conclusion section needs improvement and authors need to expand it as it will improve the quality of this study. The English level needs some improvement to reach a satisfactory level, specifically the grammar. It should sufficiently meet quality to reach scientific merit for publication. I recommend that the authors describe the study's scientific contribution to the existing body of knowledge in the discussion section. How does this study’s implications provide useful information for the scientific readership? I endorse this manuscript for publication after minor corrections, as suggested. | Thank you for your comments. We have made corrections according to the suggestions made (page 24). |
